# The influence of children’s physical activity time on the development of prosocial moral reasoning: evidence from school consolidation policy in rural northern China

**DOI:** 10.3389/fpsyg.2025.1534941

**Published:** 2025-06-20

**Authors:** Guo Dong Zhao, Zeya Bai, Yiyuan Yin, Wumin Yue, Tingting Zhang, Rui Shi, Can Guo

**Affiliations:** The Institute of Education Sciences, Shanxi University, Taiyuan, China

**Keywords:** school consolidation policy, prosocial moral reasoning, physical activity, rural education, moral development, sports participation

## Abstract

This study integrates TPSR model and Eisenberg’s theory of moral emotion as the theoretical framework to explore the influence of physical activity on prosocial moral reasoning of rural primary school students, focusing on the “school merger” policy. Data from 292 fifth and sixth-grade students in Ping Yao County were analyzed. Results showed significant correlations between physical activity frequency and prosocial moral reasoning, with physical activity negatively correlated with hedonistic tendencies and positively correlated with needs-oriented and other-oriented tendencies. Regression analysis indicated that physical activity predicted the variance in hedonistic moral reasoning (*R*^2^ = 0.184). The findings suggest that increased physical activity promotes moral reasoning development, particularly for boarders and younger students.

## 1 Introduction

With the rapid advancement of urbanization and industrialization in China, a significant number of rural residents have migrated to cities for work, resulting in a steady decline in both the permanent rural population and the number of school-age children. Consequently, many rural schools are facing a severe enrollment crisis. In response, local governments have initiated school consolidation policies, adjusting the original distribution of schools in urban and rural areas. As part of this policy, some rural schools have been closed or merged into urban schools. This restructuring has led to improved educational infrastructure and learning environments by concentrating resources. However, it has also increased the commuting distance for some rural students, necessitating boarding arrangements. As a result, many former urban schools have transitioned into mixed boarding schools.

Prosocial moral reasoning in adolescents develops in stages (Eisenberg, 1992) and is a critical dimension influencing moral behavior (Kohlberg, 1984). Due to disparities in educational resources, social interaction opportunities, and environmental factors between urban and rural settings (Murthy and Dharmayat, 2020), rural and urban children exhibit significant differences in the developmental stages of moral reasoning. Urban children may encounter complex moral situations and dilemmas earlier, fostering the development of more advanced moral reasoning. In contrast, rural children often rely more heavily on direct life experiences and traditional moral values, which may limit the diversity and depth of their moral reasoning.

Existing research suggests that participation in physical activity has a wide range of positive effects on adolescent development. First, physical activity not only contributes to physical health (Guevrement et al., 2014), but also plays a significant role in fostering prosocial behavior. Through experiences involving cooperation, decision-making, rule-following, and fair play, physical activity provide an essential platform for cultivating prosocial behavior and enhancing moral development in children (Bronikowska et al., 2020). Furthermore, physical activity have been shown to significantly promote moral reasoning by encouraging collaboration, fairness, and interpersonal skills (Bailey, 2006; Weiss, 2011).

Notably, rural and urban children differ in both the forms and frequencies of physical activity participation (Tian et al., 2021). While urban children often have access to more specialized and diverse physical activities, rural children typically engage in spontaneous, simple, and natural forms of physical activity due to limitations in facilities and resources (Marta et al., 2023). Integrating moral dilemma tasks into physical activities can also offer students practical opportunities to exercise moral reasoning, further advancing their moral development (Bronikowska et al., 2020). Pennington (2017) found that combining physical activity with moral education programs can significantly enhance children’s prosocial abilities. Certain types of physical activity, such as endurance and coordination-based physical activity, have been shown to enhance cognitive abilities like empathy (Bahmani et al., 2020), thereby promoting prosocial behavior (Moeijes et al., 2018).

At the same time, organized physical activities offer adolescents abundant opportunities for social interaction, enabling them to learn cooperation, respect, and rule compliance through communication with coaches, teammates, and opponents—thereby fostering the formation of moral norms and values (Bruner et al., 2018). For example, participation in sports clubs or team physical activity encourages athletes to work toward common goals and collaborate with one another. This not only enhances the enjoyment of physical activity but also contributes to the athletes’ holistic physical and mental development (Shields et al., 2015). Moreover, morally relevant decisions made during team physical activity—such as choosing between fair play and cheating—can have positive or negative reinforcement effects on children’s prosocial moral reasoning (Bruner et al., 2018; Kavussanu and Stanger, 2017).

On the other hand, school consolidation, as a form of organizational reform in education, also affects students’ participation in physical activity. School consolidation, often resulting in improved sports facilities and more diverse opportunities for physical activity (Howley et al., 2011). For instance, educational practices in the United States and Japan have demonstrated that school consolidation can lead to the optimization and integration of physical education resources (Nippon Bridge, 2024; Feldhoff, 2020).

Although existing studies have demonstrated the positive impact of physical activity on the development of adolescents’ prosocial behavior, little research has specifically examined how the amount of time spent on physical activity and the type of activity influence children’s prosocial moral reasoning in the context of school consolidation. This question is especially salient within the backdrop of China’s school merging policy (commonly referred to as “school closure and consolidation”) in rural areas of northern China.

In response, the present study integrates Hellison’s Teaching Personal and Social Responsibility (TPSR) model with Eisenberg’s theory of moral emotion to construct a theoretical framework. The TPSR model, proposed by Hellison (2011), emphasizes that physical education should go beyond teaching motor skills to include the development of self-control, empathy, and a sense of social responsibility through physical activity. Eisenberg’s (1986) theory of moral emotion highlights the central role of emotion in the process of moral reasoning; through physical activity participation, children experience rich emotional interactions in cooperative and competitive contexts, thereby fostering empathy and prosocial behavior.

Based on this integrated framework, we hypothesize that structured physical activity enables children to develop greater sensitivity to the needs of others through interpersonal interaction and emotional engagement, thus enhancing their prosocial moral reasoning. In the context of the rural school consolidation policy in northern China, children’s participation in physical activity not only contributes to their physical well-being but also plays a critical role in moral development—particularly in enhancing their sense of school belonging and interpersonal relationships. Therefore, this study aims to explore how physical activity supports the development of children’s prosocial moral reasoning, offering theoretical insights to inform educational practice.

## 2 Materials and methods

### 2.1 Survey and measure

The present questionnaire consists of a background information section and two thematic sub-questionnaires. The background section gathers basic demographic data, including the student’s school, grade level, gender, and family composition. The first thematic sub-questionnaire focuses on physical activity (KMO = 0.776, Cronbach’s alpha = 0.707) and aims to assess students’ engagement in physical activity. The second sub-questionnaire measures prosocial moral reasoning (KMO = 0.867, Cronbach’s alpha = 0.863) to evaluate students’ level of prosocial moral development. Overall, the questionnaire demonstrated good reliability and validity, with a total KMO of 0.817 and Cronbach’s alpha of 0.766.

Physical activity was assessed using the PAQ-C (Physical Activity Questionnaire for Older Children), as outlined in the Physical Activity Questionnaire for Older Children (PAQ-C) and Adolescents (PAQ-A) Manual. The PAQ-C is designed for children in Grades 4 to 8 (approximately 8–14 years old) who are currently enrolled in school and engage in regular weekly physical activity. The instrument has been used among Chinese youth and has demonstrated good reliability and validity. In the current study, the Cronbach’s alpha was 0.707, indicating satisfactory internal consistency. The PAQ-C contains seven items that assess the types of physical activity students participated in over the past 7 days, their level of engagement in physical education classes, and the intensity and frequency of their physical activity. Each item is scored on a 5-point Likert scale, and the overall physical activity score is derived from these responses.

Specifically, Item 1 generates a composite score based on the average frequency of all listed activities (1 point for “none” to 5 points for “7 times or more”). Items 2 through 5 are rated on a 5-point scale, with Level 1 representing the lowest engagement (1 point) and Level 5 the highest (5 points). Item 6 records the average daily activity frequency over the past week, scored from 1 (“no activity”) to 5 (“as much activity as possible”). Item 7 is used to identify participants with atypical activity patterns during the week but is not included in the total physical activity score. The final physical activity score is computed by averaging the scores of the first six items, with a score of 1 indicating low physical activity and a score of 5 indicating high physical activity.

Prosocial moral reasoning was assessed using the Prosocial Moral Reasoning Objective Measure (PROM) developed by Carlo et al. (1992). The PROM is based on Rest’s Defining Issues Test (DIT) (Rest, 1963) and has proven effective among adolescents aged 13–21+. It has also been validated in studies involving Chinese youth, consistently demonstrating good reliability, convergent validity, and discriminant validity. In this study, the Cronbach’s alpha was 0.863.

The PROM includes three moral dilemma stories. Participants are asked to read each scenario and assist the protagonist in making a moral decision. Each story contains five items representing five levels of prosocial moral reasoning: hedonistic, approval-oriented, needs-oriented, stereotyped, and internalized reasoning. After making a decision, participants rate the influence of each reasoning type on their decision using a 5-point scale (1 = not influential at all, 5 = very influential). Each story also includes a lie-detection item to identify inattentive or random responders. In this study, participants’ scores on the internalized reasoning subscale (representing the highest level of moral reasoning) were used as an indicator of their prosocial moral reasoning ability. Higher scores reflect a higher level of moral reasoning.

To ensure the appropriateness and validity of the questionnaire, several items were carefully revised and optimized based on regional and school-specific characteristics, while maintaining the original structure and intent of the instrument. As a preliminary validation step, an analysis was conducted on a sample of 294 responses to assess the questionnaire’s applicability and accuracy.

The sample data analyzed in this study were collected through an in-person survey on physical activity and prosocial behavior conducted on May 28, 2024, at Xiangyuan Primary School in Pingyao County, Jinzhong City, Shanxi Province. The survey targeted students in Grades 5 and 6. A total of 302 questionnaires were distributed, and after excluding responses with abnormal activity patterns identified through Item 7 of the PROM, a final dataset of 294 valid questionnaires was retained. Among the valid responses, 172 were from fifth-grade students (90 boys, 82 girls), and 122 were from sixth-grade students (56 boys, 66 girls).

### 2.2 Analysis

This study utilized SPSS 27.0 and jamovi 2.5.6 to conduct an in-depth analysis of the physical activity frequency and prosocial moral reasoning levels of 294 valid samples from fifth- and sixth-grade students. Descriptive statistics were used to examine the distribution of basic characteristics such as gender and boarding status, and to assess the average levels and variability in students’ physical activity frequency and various dimensions of prosocial moral reasoning.

Correlation analysis was conducted to explore the relationship between physical activity frequency and prosocial moral reasoning levels. Further, independent samples t-tests were employed to examine whether gender and boarding status had significant effects on physical activity frequency and prosocial moral reasoning. Finally, regression analysis was conducted to investigate the influence of weekly physical activity frequency, weekend activity frequency, and extracurricular physical activity frequency on scores related to hedonistic reasoning, as well as the impact of weekly physical activity frequency on need-oriented reasoning scores.

## 3 Result

### 3.1 Descriptive analysis

This study conducted a descriptive analysis to understand the distribution characteristics of the sample. A total of 294 valid student responses were collected. Among them, 46 students were boarders, accounting for 15.6% of the sample, while 248 students were non-boarders, making up 84.4%. Regarding gender distribution, there were 146 boys (49.7%) and 148 girls (50.3%), as shown in Table 1.

**Table 1:** Sample statistics (*n* = 294).

Category		Grade 5	Grade 6	Total
Boarding	Count	20	26	46
	% Within Boarding Status	43.5%	56.5%	100.0%
	% Within Grade	11.6%	21.3%	15.6%
Non-boarding	Count	152	96	248
	% Within Boarding Status	61.3%	38.7%	100.0%
	% Within Grade	88.4%	78.7%	84.4%
Male	Count	90	56	146
	% Within Gender	61.6%	38.4%	100.0%
	% Within Grade	52.3%	45.9%	49.7%
Female	Count	82	66	148
	% Within Gender	55.4%	44.6%	100.0%
	% Within Grade	47.7%	54.1%	50.3%

### 3.2 Correlation analysis

The study conducted a correlation matrix analysis for all variables, as shown in Table 2. The means (M) and standard deviations (SD) reflect general tendencies and individual differences among students on the variables. Students participated in physical activities about 2 times on average in the past 7 days, with relatively low variability in participation frequency (*M* = 2.312, SD = 0.686). They demonstrated a high level of enthusiasm for physical education classes (*M* = 4.360, SD = 1.014). On average, students exercised approximately 3 days per week after school, with relatively greater variability in physical activity days (*M* = 3.160, SD = 1.117).

**Table 2:** Means, standard deviations, and correlation coefficients of variables.

Variable	Mean	SD	1	2	3	4	5	6	7	8	9	10	11	12
1. Frequency of Physical Activity	2.3124	0.686	1	0.201^**^	0.189^**^	0.275^**^	0.364^**^	0.429^**^	−0.120^*^	0.035	0.035	−0.006	0.039	0.550^**^
2. Enthusiasm for physical education Classes	4.360	1.014		1	0.047	0.266^**^	0.259^**^	0.243^**^	−0.166^**^	0.034	0.048	0.013	0.053	0.415^**^
3. Days of Physical Activity After School	3.160	1.117			1	0.399^**^	0.374^**^	0.316^**^	−0.075	−0.040	0.119^*^	0.020	−0.019	0.565^**^
4. Weekend Physical Activity Frequency	3.730	0.990				1	0.413^**^	0.333^**^	−0.166^**^	0.011	0.080	0.026	0.036	0.657^**^
5. Physical Activity Frequency During Leisure Time	3.290	0.995					1	0.460^**^	−0.178^**^	−0.008	0.126^*^	0.146^*^	−0.069	0.665^**^
6. Weekly Physical Activity Frequency	3.111	0.682						1	−0.169^**^	0.195^**^	0.024	−0.046	−0.037	0.640^**^
7. Hedonistic Score	0.196	0.021							1	−0.247^**^	−0.281^**^	−0.185^**^	−0.198^**^	−0.237^**^
8. Needs-Oriented Score	0.206	0.024								1	−0.288^**^	−0.193^**^	−0.371^**^	0.110
9. Approval-Oriented Score	0.198	0.022									1	−0.137^*^	−0.257^**^	0.070
10. Stereotype Score	0.195	0.019										1	−0.316^**^	0.022
11. Internalization Reasoning Score	0.205	0.025											1	0.011
12. Average Physical Activity Engagement	3.152	0.541												1

**Correlation is significant at the 0.01 level (two-tailed).

*Correlation is significant at the 0.05 level (two-tailed).

The results indicate that the time invested in physical activity is correlated with prosocial moral reasoning, mainly reflected in five dimensions:

Correlation between physical activity time investment and hedonistic tendencies in prosocial moral reasoning: The frequency of physical activity in the past 7 days, enthusiasm for physical education classes, weekend physical activity frequency, leisure time physical activity frequency, weekly physical activity frequency, and average physical activity engagement were all significantly negatively correlated with the hedonistic score in the first stage of moral reasoning. This suggests that with increased time invested in physical activity, students tend to show lower hedonistic tendencies in moral reasoning — meaning they do not simply pursue immediate pleasure and satisfaction.

Correlation between physical activity time investment and needs-oriented tendencies in prosocial moral reasoning: Weekly physical activity frequency was significantly positively correlated with the needs-oriented score (*r* = 0.195). This indicates that students who regularly participate in physical activity are more likely to consider the needs and interests of others in moral decision-making. This finding supports the positive role of physical activity in cultivating needs-oriented prosocial moral reasoning.

Correlation between physical activity time investment and approval-oriented tendencies in prosocial moral reasoning: physical activity days after school and leisure time physical activity frequency showed significant positive correlations with approval-oriented scores. This suggests that the more time students invest in physical activity, the more likely they are to consider others’ approval and recognition in their moral reasoning.

Correlation between physical activity time investment and stereotype tendencies in prosocial moral reasoning: Leisure time physical activity frequency was significantly positively correlated with stereotype scores. This may indicate that as students increase their frequency of physical activity, their tendency to rely on stereotypes in moral reasoning also rises.

Correlation between physical activity time investment and internalization tendencies in prosocial moral reasoning: No significant correlation was found between physical activity time investment and internalization tendencies. This may suggest that the influence of physical activity on internalized moral reasoning is limited, or that internalized reasoning is more influenced by individual values, beliefs, and educational background.

### 3.3 Difference analysis

Difference analyses of prosocial moral reasoning ability and physical activity time investment were conducted from the perspectives of gender, boarding status, and grade level, as shown in Tables 3–5.

**Table 3:** Difference analysis of hedonism scores by gender.

Main variable	Gender	M ± SD	*t*	*p*
Hedonism Score	Male	0.193 ± 0.021	−2.828	0.005
	Female	0.199 ± 0.020		0.005

**Table 4:** Difference analysis of physical activity time investment by boarding status.

Main variable	Boarding status	M ± SD	*t*	*p*
Weekly frequency of physical activity	Boarding	2.843 ± 0.591	−2.940	0.004
	Non-boarding	3.161 ± 0.687		
Frequency of participation in physical activities	Boarding	1.933 ± 0.615	−4.203	<0.001
	Non-boarding	2.383 ± 0.676		

**Table 5:** Analysis of differences in physical activity time investment and weekly physical activity frequency by grade level.

Main variable	Grade	M ± SD	*t*	*p*
Frequency of extracurricular physical activity	Grade 5	3.400 ± 0.921	2.230	0.027
	Grade 6	3.130 ± 1.076		
Weekly physical activity frequency	Grade 5	3.178 ± 0.661	2.035	0.043
	Grade 6	3.014 + 0.702		

First, regarding the difference analysis of moral reasoning ability by gender, there was a significant difference in hedonic reasoning between males and females (*t* = −2.087, *p* = 0.005). Boys had a lower hedonic reasoning score (*M* = 0.193) compared to girls (*M* = 0.199), indicating that, given the same level of physical activity time investment, girls tend to pursue more immediate satisfaction and short-term pleasure than boys.

Second, in the difference analysis of physical activity time investment by boarding status, there was a significant difference in the frequency of physical activity participation during the week. Specifically, boarding students had a lower average frequency of physical activity during the week (*M* = 2.843) compared to non-boarding students (*M* = 3.161), and this difference was statistically significant (*t* = −2.940, *p* = 0.004). Additionally, the number of times students participated in physical activity also differed significantly by boarding status, with boarding students participating fewer times (*M* = 1.933) than non-boarding students (*M* = 2.383), and this difference was also statistically significant (*t* = −4.203, *p* < 0.001).

Third, regarding the differences in physical activity time investment across grade levels, both extracurricular physical activity frequency and weekly physical activity frequency show significant differences. The data indicate that fifth-grade students have higher extracurricular activity frequency (*M* = 3.400) and weekly physical activity frequency (*M* = 3.178) compared to sixth-grade students, and these differences are statistically significant.

### 3.4 Regression analysis

Based on the results of the above data analysis, a regression analysis was conducted using the variables related to hedonistic prosocial moral reasoning scores—namely, students’ frequency of participation in physical activity, enthusiasm for physical educationclasses, number of weekend physical activity sessions, number of after-school physical activity days, and extracurricular physical activity frequency—as independent variables. The results revealed that time investment in physical activity had a significant multiple linear regression relationship with hedonistic prosocial moral reasoning ability, as shown in Table 6.

**Table 6:** Multiple linear regression of time investment in physical activity on hedonistic prosocial moral reasoning.

Variable	*B*	*β*	*t*	*p*	*F*	Adjusted R^2^
Extracurricular physical activity frequency (Item 5–1)	−0.025	−0.012	−2.165	0.033	2.060	0.233
After-school physical activity days (Item 4–1)	−0.167	−0.008	−0.499	0.036		
Enthusiasm for physical education class (Item 2–1)	0.018	0.015	1.196	0.235		
Frequency of participating in physical activity	−0.002	0.003	−0.900	0.370		
Weekly frequency of physical activity	0.003	0.003	1.000	0.319		

The results show that the model explains 23.3% of the variance in the dependent variable and is statistically significant overall (*F* = 2.060, *R*^2^ = 0.233, *p* = 0.021). Since most of the participants are at an age where hedonistic moral reasoning is predominant, this regression model (i.e., Table 6) only accounts for part of the variance in the hedonistic dimension of moral reasoning.

The intercept is significant, indicating that when all predictor variables are at their reference levels, the expected value of the dependent variable is 0.207. Among the predictors, the “5–1” level of extracurricular physical activity frequency (*β* = −0.012, *p* = 0.033) and the “4–1” level of after-school physical activity days (*β* = −0.008, *p* = 0.036) have significant negative effects on hedonistic prosocial moral reasoning, while the effects of the other variables are not significant.

Results from collinearity analysis and normality tests indicate that the model meets the basic assumptions of linear regression and that there are no autocorrelation issues. Therefore, we conclude that the more time students invest in physical activity—especially in terms of extracurricular physical activity frequency and after-school physical activity days—the lower their tendency toward hedonistic reasoning in prosocial moral decision-making.

## 4 Discussion

This study employed descriptive statistics, correlation analysis, difference analysis, and regression analysis to explore the impact of time investment in physical activity on the development of prosocial moral reasoning among rural primary school students in North China under the school consolidation policy. The results revealed several key findings, providing empirical evidence for moral education in primary schools.

The study found a significant negative correlation between time investment in physical activity and the hedonistic tendency in prosocial moral reasoning. This indicates that increased frequency and intensity of physical activity help reduce children’s reliance on short-term gratification in moral decision-making. This finding aligns closely with the research of Kavussanu and Roberts (2001), who similarly found that children participating in physical activities demonstrated more principled thinking in moral judgment. Furthermore, the results echo the classic perspective of Opstoel et al. (2020), which holds that physical activity can effectively foster prosocial behavior by offering moral practice scenarios. However, the findings of this study differ in some respects from those of Bandura et al. (1996), who emphasized the prevalence of moral disengagement in competitive physical activity. In contrast, this study found that physical activity overall promotes the development of moral reasoning. This discrepancy may be due to differences in the age of the study participants (this study focused on children) and the types of physical activity involved (this study included more cooperative physical activities).

Further analysis revealed that gender, boarding status, and grade level had significant effects on prosocial moral reasoning. With the same level of time investment in physical activity, girls exhibited a higher tendency toward hedonism, which may reflect gender differences in emotional expression and perceptions of immediate gratification during the socialization process. In addition, boarding students exercised significantly less frequently than non-boarding students, possibly due to school management concerns such as safety, which limited boarding students’ time for physical activity. Finally, grade-level differences showed that fifth-grade students had higher participation in physical activity than sixth-grade students, which may be attributed to the increased academic pressure faced by students in higher grades.

Due to regional constraints, we are unable to determine from the data whether other potential influencing factors exist, and our findings still lack qualitative analysis of children’s prosocial moral development. Nevertheless, this study highlights the dual impact of the rural school consolidation policy on optimizing the physical activity environment and promoting children’s moral development. Following the implementation of this policy, government funding for rural schools could be more concentrated, which has, to some extent, improved sports facilities in these schools. However, with the increased adoption of boarding systems, school management has become more collectivized, resulting in restricted time for physical activity for some students and consequently affecting their participation in physical activity.

Future research should further explore the internal mechanisms linking physical activity and moral development, particularly the roles of different types of physical activity and environmental factors. It is important to tailor physical activity programs and schedules based on students’ gender, boarding status, and grade level to better support moral development and to provide rural schools with more targeted physical activity solutions.

Under the context of China’s rural school consolidation policy, this study analyzed the impact of time investment in physical activity on children’s prosocial moral reasoning. The results indicate that increased physical activity time significantly enhances students’ levels of prosocial moral reasoning, particularly in dimensions such as need-oriented and approval-oriented reasoning. The school consolidation policy has facilitated more centralized investment in education by the government, enabling rural schools to be equipped with better sports facilities and providing an improved environment for physical activity, which in turn effectively fosters students’ moral development.

Given the relatively low levels of physical activity participation among boarding students and upper-grade primary school students in rural areas, schools should focus on designing targeted physical education curricula and activities tailored to students in boarding environments and different grade levels. It is also crucial to improve the construction of sports facilities, encourage diverse physical activity participation, and allocate dedicated time for physical activity.

Overall, time investment in children’s physical activity plays a positive role in the development of prosocial moral reasoning. To further support the comprehensive and healthy moral development of children, collaborative efforts among the government, families, and schools are essential to ensure that children have sufficient and diverse opportunities for physical activity. Future research could focus on the types of physical activities and specific environmental factors to deepen our understanding of the relationship between physical activity and moral reasoning, thereby offering more scientifically grounded guidance for moral education in children.

## Data Availability

The raw data supporting the conclusions of this article will be made available by the authors, without undue reservation.
